# Transcriptome analysis of four types of gonadal tissues in largemouth bass (*Micropterus salmoides*) to reveal its sex-related genes

**DOI:** 10.3389/fgene.2024.1459427

**Published:** 2024-08-26

**Authors:** Dongyun Zhang, Taihang Tian, Shengjie Li, Jinxing Du, Caixia Lei, Tao Zhu, Linqiang Han, Hongmei Song

**Affiliations:** ^1^ Key Laboratory of Tropical and Subtropical Fishery Resource Application and Cultivation, China Ministry of Agriculture, Pearl River Fisheries Research Institute, Chinese Academy of Fisheries Sciences, Guangzhou, China; ^2^ College of Life Science, Huzhou University, Huzhou, Zhejiang, China; ^3^ College of Fisheries and Life Science, Shanghai Ocean University, Shanghai, China; ^4^ Guangdong Province Liangshi Aquaculture Seed Industry, Foshan, Guangdong, China

**Keywords:** LMB, sex reversal, gonadal transcriptome, DEGs, 17α-methyltestosterone, 17β-estradiol

## Abstract

The sex determination system of largemouth bass (*Micropterus salmoides*, LMB) is XX/XY; however, the underlying molecular mechanisms involved in early sex differentiation, gonadal development, and exogenous hormone-induced sex reversal remain unknown. In this study, LMB at 15 days post-hatching (dph) were fed diets containing 20 mg/kg of 17α-methyltestosterone (17α-MT) or 30 mg/kg of 17β-estradiol (17β-E_2_) for 60 days, respectively. Serum steroid levels, histological observations of the gonads, and identification of sex-specific markers were employed to screen the gonads of 60-day-old normal female fish (XX-F), normal male fish (XY-M), 17β-E_2_ induced pseudo-female fish (XY-F), and 17α-MT-induced pseudo-male fish (XX-M) for transcriptome sequencing in order to uncover genes and pathway involved in the process of sexual reversal. The results from histology and serum sex steroid hormone analysis showed that both 17α-MT and 17β-E2 were capable of inducing sex reversal of LMB at 15 dph. Transcriptome results revealed a total of 2,753 genes exhibiting differential expression, and the expression pattern of these genes in the gonads of XX-M or XY-F resembled that of normal females or males. The male sex-biased genes that are upregulated in XX-M and downregulated in XY-F are referred to as key genes for male reversal, while the female sex-biased genes that are upregulated in XY-F and downregulated in XX-M are referred to as key genes for female reversal. Finally, 12 differentially expressed genes (DEGs) related to male sex reversal were screened, including *star2*, *cyp17a*, *cyp11b1*, *dmrt1*, *amh*, *sox9a*, *katnal1*, *spata4*, *spata6l*, *spata7*, *spata18* and *foxl3*. 2 DEGs (*foxl2a* and *cyp19a1b*) were found to be associated with female sex reversal. The changes in these genes collectively influence the direction of sex differentiation of LMB. Among them, *star2*, *dmrt1* and *cyp19a1b* with significantly altered expression levels may play potentially crucial role in the process of gender reversal. The expression patterns of 21 randomly selected genes were verified using qRT-PCR which confirmed the reliability and accuracy of the RNA-seq results. These findings not only enhance our understanding of the molecular basis underlying sex reversal but also provide crucial data support for future breeding research on unisexual LMB.

## Introduction

The largemouth bass (*Micropterus salmoides*, LMB), a member of the Perciformes order and Centrarchidae family, belongs to the genus *Micropterus*. It possesses desirable meat quality, lacks intermuscular spines, exhibits rapid growth, and has a short cultivation cycle. Consequently, it is highly favored by both consumers and breeders alike, making it an economically significant freshwater aquaculture species (Bai and Li, 2013). In recent years, the breeding area of LMB in China has been expanding and gradually moving towards the north and inland. During the breeding process, female LMB allocate a substantial amount of energy towards ovarian development, which not only increased breeding costs but also limits commercial production needs (Lu et al., 2022). The lack of improved species has become one of the important factors that restrict the development of the entire aquaculture industry (Gui, 2005). Generally, male LMB grow faster than females after 270 dph (days post hatching, dph) (Wei, 2023). This makes the production of all-male LMB seedlings highly valued in aquaculture. Previously, pseudo-females induced by 17β-E_2_ (50–100 mg/kg) and pseudo-males induced by 17α-MT (50–100 mg/kg) were successfully obtained in LMB using sex-specific molecular markers and hormone-induced sex reversal techniques (Zhou, et al., 2021; Du et al., 2021; Du et al., 2022). Subsequently, we tried to induce sexual reversal in LMB at lower concentrations of 30 mg/kg 17β-E_2_, and found that this concentration could successfully induce sexual reversal in LMB (Zhang, et al., 2024). The lower concentration of 17α-MT (20 mg/kg) also could obtain pseudo-males in LMB (Unpublished data). However, our understanding of the mechanism underlying sex determination and differentiation in LMB remains limited, with unknown key genes involved in sexual reversal and theoretical research lagging behind production practice.

Mammals determine sex through various genes on the Y chromosome, while teleost fish lack a unified sex chromosome and have randomly distributed genes involved in sex determination (Katsura et al., 2018). At present, some sex-related genes in fish have been reported. For example, *dmrt1* is involved in the development and maintenance of testes in medaka (*Oryzias latipes*) (Masuyama et al., 2012); deletion of *amh* gene leads to male-to-female differentiation in nile tilapia (*Oreochromis niloticus*) (Eshel et al., 2014); while *sdy* is considered a major sex gene in rainbow trout (*Oncorhynchus mykiss*), its overexpression in females results in the appearance of testes (Yano et al., 2012). In LMB, sex-related genes have been reported, for example, *dmrt1* was highly expressed in the testis (Yan et al., 2019); *amh* may be involved in early testis development (Li et al., 2023). However, the mechanism of sex differentiation still needs further analysis.

Transcriptomics is the investigation of comprehensive RNA expression patterns in specific cells or organisms, which facilitates understanding of gene expression dynamics during various developmental stages, diverse environmental conditions, and inter-gene regulatory mechanisms (Li and Li, 2014). Using transcriptome sequencing (RNA-seq) technology to explore teleost fish gonads can provide invaluable insights into the molecular mechanism underlying gonadal development and reproductive process (Abdelrahman et al., 2017). In recent years, RNA-seq technology has revealed numerous differentially expressed genes related to sex differentiation and development in fish. For instance, in helmet catfish (*Cranoglanis bouderius*), the genes *amh*, *dax1*, *nanos1* and *ar* played important roles in the differentiation and development of the testis (Wang et al., 2022). On the other hand, *foxl2*, *sox3*, *foxo*, *zar1* and *cyp19a1* exhibited high expression levels in the ovaries of paradise fish (*Macropodus opercularis*), while *cyp11b*, *amh*, *dmrt2*/3, *sf1*, *sox9*, *gdf6*, *fstl1* and *hsd11b2* were specifically expressed in the testes (Liu F. et al., 2023). Additionally, Dan et al. (2018) proposed that *pfpdz1* played a key role in sperm development of yellow catfish (*Pelteobagrus fulvidraco*). In conclusion, the identification of candidate genes involved in sex determination and differentiation in teleost fish can be inferred by RNA-seq technology with histological observation, enabling effective screening of key genes governing sex differentiation.

In this study, RNA-seq technology was used to screen differentially expressed genes and pathways in the gonads of normal females, normal males, pseudo-females, and pseudo-males in LMB. It analyzed the genes that changed during sex reversal, identified the genes related to sex differentiation and sex reversal, observed the changes in gonadal tissue structure, and analyzed the levels of sex steroid hormones. This will provide valuable resources for elucidating the molecular mechanisms of underlying sex differentiation and gonadal development (including hormone-induced sexual reversal) of LMB, ultimately, contributing to monosexual LMB production and enhancing aquaculture productivity.

## Materials and methods

### Preparation of experimental feed and feeding management

The LMB used in the experiment were sourced from Guangdong Liangshi Aquaculture Co., LTD. and reared in a circulating aquaculture system with a diameter and depth of 1 m each. Each aquaculture tank housed 1000 LMB (10.0 ± 0.1 mm, 15 days post-hatching (dph)). The experimental compounds, namely, 17β-E_2_ (MCE#CGS20267) and 17α-MT (MCE#HY-A0121), were procured from Guangzhou Weike Biotechnology Co., LTD. The fish were divided into three groups: control group, 17α-MT treatment group receiving a dosage of 20 mg/kg (M20), and 17β-E_2_ treatment group receiving a dosage of 30 mg/kg (E30). The diets for each group were prepared using the spray method described by El-Greisy and El-Gamal. (2012), air-dried, stored in cool and dark conditions. The control group’s diet was treated with anhydrous ethanol devoid of 17α-MT or 17β-E_2_. Feeding the hormone-containing diet lasted for a duration of 60 days, with full meals provided three times daily at 8:00, 12:00, and 17:00, respectively. After feeding, the remaining bait and debris were promptly removed. Subsequently, the entire experimental fish was transferred to a cement tank (7 m × 3 m × 1.2 m) and fed specialized bass diets until 120 dph, and water changes were performed regularly, once a week, by a third each time.

### Sampling and analysis

Based on previous molecular markers for sex determination, a single band of 464 bp was amplified in female LMB, while male LMB exhibited double bands (406 bp and 464 bp) (Du et al., 2021). Consequently, the genotype of an individual can be ascertained by the number and size of these amplified bands. After being fed a hormone-containing diet for 60 days, 30 fish were randomly selected from each group and anesthetized with 100 mg/L MS-222 (Sigma, MO, United States). The peritoneal membrane attached to the gonad tissue was removed and a portion of it was immediately stored in liquid nitrogen for total gonad RNA extraction. Another part was fixed in a 4% paraformaldehyde solution for preparation of gonad tissue sections. Additionally, an appropriate amount of caudal fin was clipped and stored in anhydrous ethanol for genetic gender identification. Subsequently, the samples were categorized into four types based on the results of genetic sex identification and gonadal tissue sections: normal female fish (XX-F), normal male fish (XY-M), 17β-E_2_ induced pseudo-female fish (XY-F), and 17α-MT-induced pseudo-male fish (XX-M). Three samples were taken from each group, with each sample consisting of a mixture of three gonadal tissues for RNA-seq sequencing. To validate the accuracy of RNA-seq sequencing results, qRT-PCR verification was performed using four gonad tissue samples as described above. Blood samples (n = 9) from all four species at 120 dph were extracted, left at 4°C for 3 h before centrifugation at 3,000 rpm for 15 min using a low temperature centrifuge to isolate serum which was then stored at −80°C refrigerator to measure estradiol (E_2_) and testosterone (T) content levels. All experiments involving fish followed the guidelines for the Care and Use of Laboratory Animals approved by China Science Foundation’s Laboratory Animal Committee.

### Preparation of HE tissues sections

The gonadal tissue fixed in 4% paraformaldehyde were dehydrated with gradient alcohol, transparentized with benzyl alcohol, embedded using an embedding machine, and then observed and photographed using a ZEISS microscope (Axio Scope.A1) after sectioning and hematoxylin-eosin staining.

### Detection of serum E_2_ and T concentrations

The gender markers of LMB were used to identify XY-F and XX-M in the experimental groups, as well as XX-F and XY-M in group control. E_2_ and T levels were detected following the instructions provided by the Fish Estradiol (E_2_) and Testosterone (T) ELISA Kit. The optical density (OD) was determined at 450 nm with a spectrophotometer (Biotek Cytation5, United States). Subsequently, the concentrations of E_2_ and T in the samples were determined by comparing their O.D. values to the standard curve.

### Transcriptome sequencing and analysis

The total RNA was isolated from the gonads of XX-F, XY-M, XY-F and XX-M using the TRIzol^®^ kit (Invitrogen, United States) according to the manufacturer’s protocol. The concentration and purity of RNA were determined by Nanodrop 2000 (Thermo Scientific, Delaware, United States). RNA quality was assessed by 1% agarose gel electrophoresis and Agilent2100. All samples had OD260/OD280 ratios ranging from 1.8 to 2.1 indicating high-quality RNA with integrity values ≥ 8.0 for all samples. A total of 12 samples were used for the construction of sequencing libraries. Magnetic beads with Oligo (dT) were employed to isolate mRNA from total RNA which was subsequently fragmented into small fragments (∼300 bp) using a fragmentation buffer. The first-strand cDNA synthesis involved random primers, followed by second-strand cDNA synthesis and “A” tailing with attached sequencing adaptors. After undergoing various purification steps including adaptor product purification, fragment sorting, and PCR amplification, we successfully obtained the final library. High-throughput Illumina NovaSeq6000 sequencing will be employed to generate sequence data for these libraries which have already been submitted to GenBank (https://submit.ncbi.nlm.nih.gov/subs/sra, SRA login number: PRJNA1060465). To ensure the quality and accuracy of raw reads in each sample, default parameters provided by FASTP software were utilized for data quality and accuracy assessment (Chen et al., 2018). Clean reads were obtained after removing adapter sequences and low-quality reads during preprocessing steps using HISAT2, a second-generation sequencing alignment software tool, against a reference genome (https://www.ncbi.nlm.nih.gov/datasets/genome/GCF_014851395.1/) (Kim et al., 2015). New transcripts and genes were identified employing String Tie software based on the mapping results obtained through assembly and alignment processes (Mihaela et al., 2015). Gene expression levels were quantitatively analyzed using RSEM software based on TPM (Transcripts Per Million reads) values calculated for each gene transcript (Dewey and Li, 2011; Love et al., 2014). DEGSeq2 software was then used to analyze the differentially expressed genes (DEGs) between samples, with a threshold was set p-adjust<0.05 and |Log_2_(Fold Change) |>1. GO functional enrichment analysis and KEGG pathway analysis of differential genes using GOATOOLS and KOBAS software (Xie et al., 2011).

### qRT-PCR validation and statistical analysis of data

Twenty-one genes related to sex development were selected for data verification, and the necessary primers were synthesized by Shanghai Sangong Biological Engineering Co., LTD (Table 1). Sex-related genes screened from RNA-seq were validated in other sex reversal fish of the same age, and the information of the required primers were shown in Supplementary Table S1. *β-actin* was used as the internal reference gene (Zhou et al., 2021), the qRT-PCR reaction system was performed as follows: 50°C 2 min, 95°C 2 min (95°C 15 s, 60°C 30 s, 72°C 30 s, 40 cycles), 95°C 15 s, 60°C 1 min, 95°C 15 s. The relative expression levels of target genes were calculated using 2^-△△CT^ method, and the data were expressed as Mean ± SD. One-Way ANOVA significance analysis was conducted using IBM SPSS Statistics 25.0 software, and GraphPad Prism 9.0 was used for mapping.

**TABLE 1:** Primers used in qRT-PCR verification.

Gene	Forward primer sequence (5′-3′)	Reverse primer sequence (5′-3′)
*bmp8a*	CCC​CAG​CAT​CCA​TAA​TCG​G	TGC​CTT​TGG​AAC​CTC​ATC​G
*dmrt2a*	AGG​ACT​CAT​CAG​ACC​CTC​AAC​G	TGT​TGG​GAA​TGA​GAG​TTG​TTG​TG
*star2*	TCT​GCT​GTG​GAA​TCT​CCT​ACC​C	GAG​CCT​TTC​TCA​TTG​CTT​CTT​G
*sox9*	GTG​AAG​AAC​GGA​CAG​AGC​GAG​T	TAG​GTC​CAT​CTT​GCC​TGA​ACT​G
*amh*	CCA​GTT​GGA​GAA​GGA​GAA​GAA​GGT	CTG​GGG​AAA​CAG​GAA​CTG​ATA​CAA
*dmrt1*	GAA​CCA​CGG​CTA​TGT​GTC​TCC​T	AAT​CCC​AAG​TTC​CTC​CTC​CTG​A
*tdrd7a*	AGC​AGG​CCT​CCA​TGT​TAT​TC	GTT​CCC​ACT​GCT​CAC​CTT​TA
*esr1*	GCG​GCG​TCT​CCT​CTT​CAG​TC	GGA​CTC​CTG​GAC​CTG​TGG​GT
*foxo3b*	AGG​AAA​GCA​TCA​TCT​CGT​CGT​AA	GCT​GTT​ACT​GTC​GCC​TTT​GTC​TT
*cyp7b*	CTG​GAA​GAA​CGG​CAG​CGT​GTA​C	CTT​GAT​GAA​CTG​TGA​AGT​GTT​GGA​C
*rspo1*	GAG​ACG​GCA​AAG​ATG​GCA​GTA​GA	GTG​ATG​CTG​GAA​ACA​GTG​GTG​GT
*zar1*	GTG​CCT​ATG​TTT​GGT​GTG​TTC​AG	AGC​GTG​CTT​TGT​TAC​AAG​TGT​GG
*hsd17b12a*	GAC​CAG​GAC​CAT​TGC​AGT​TGA	TCG​GGG​TAA​GGG​TAG​GAC​ACA
*foxl2a*	CGGAGGATGACGCAATGG	GGTGGTTTCTGGGACGGA
*hsd3b1*	TTA​TGT​GGG​CAA​CGT​GGC​TAT	CGG​TGG​GAC​AAT​GCG​TAT​GA
*cyp11a*	TGA​AGT​TGG​TGA​GGA​TTT​TGT​GG	AGG​AGG​TAT​GTA​GAG​CAT​GGG​TG
*plk1*	GCA​AAT​GGG​TGG​ACT​ACT​CTG​AC	AGA​GCG​TAT​GCT​GAG​GTA​GGA​TT
*wnt4*	TGC​GAC​TAT​GAC​CCA​CGC​TCC​C	TCC​ACC​ATT​TTG​CGG​CAC​TGT​T
*katnal1*	AGC​CAG​TTG​ACA​TCC​TTG​CC	AAC​CCC​TTT​CTT​TCC​CTT​ATC​A
*cyp19a1b*	GCC​ATC​CTA​GTC​GCT​CTG​TTG​TC	TGT​TGG​TCT​GCC​TGA​TGC​TGT​TG
*sox7*	TGA​GGT​GGA​CCG​CAA​TGA​GTT​T	GAG​CAC​GGA​AAT​GAG​GCT​GGT​T

## Results

### Histological structure of gonad of LMB

**FIGURE 1:**
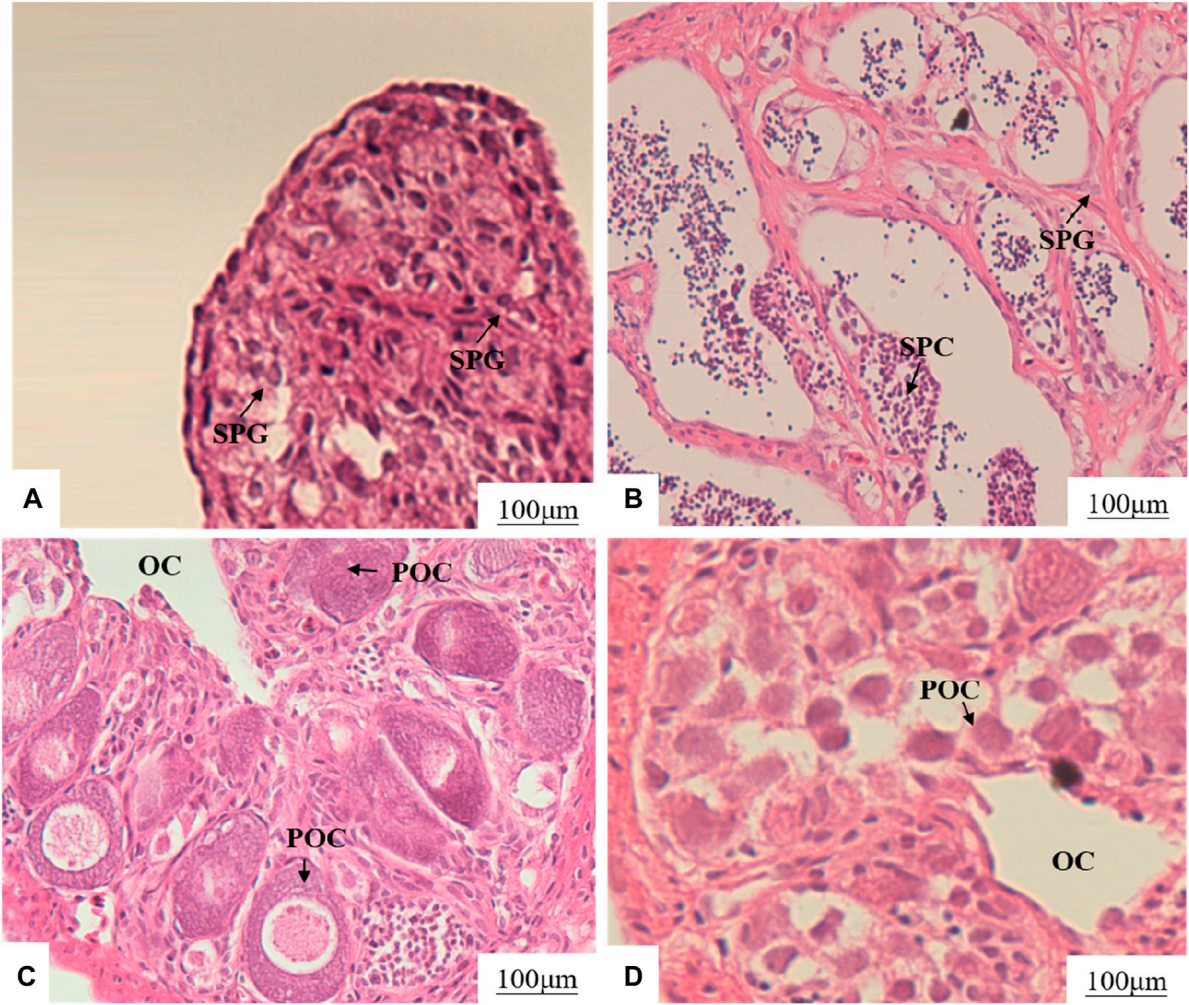
Effects of MT and 17β-E_2_ on gonadal development of 75 dph LMB. **(A)**: XY-M; **(B)**: XX-M; **(C)**: XX-F; **(D)**: XY-F. SPG, spermatogonia; SPC, spermatocytes; OC, ovarian cavity; POC, primary oocytes.

The section of gonad tissue revealed an even distribution of spermatogonia and interstitial tissue in the testes of XY-M (Figure 1A). In the testes of XX-M, spermatocytes were present with a small amount of surrounding interstitial tissue (Figure 1B). The XX-F and XY-F both showed the presence of primary oocytes and ovarian cavity (Figures 1C, D).

### Concentration of E_2_ and T in serum of 120dph LMB

**FIGURE 2:**
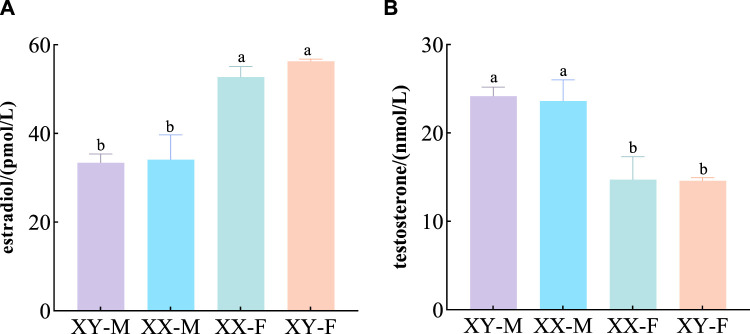
Effects of 17α-MT and 17β-E_2_ on E_2_ **(A)** and T **(B)** concentrations in 120 dph LMB.

The concentration of E_2_ and T in serum indicated that there was no significant difference in E_2_ between XY-F and XX-F (*p* > 0.05), but it was significantly higher than that in XX-M and XY-M (*p* < 0.05) (Figure 2A). There was no significant difference in T content between XX-M and XY-M (*p* > 0.05), but it was significantly higher than that in group XY-F and XX-F (*p* < 0.05) (Figure 2B).

Different letters between the treatments in the same period show significant difference (*p* < 0.05), the same letter indicates that the difference is not significant (*p* > 0.05).

### Gonads transcriptome sequencing

Fastp software was used to eliminate low-quality reads using default parameters, resulting in 7.11, 7.90, 7.21 and 7.70 Gb of valid data obtained from the gonads of XX-M, XY-F, XY-M, and XX-F samples respectively. Consequently, a total of 29.92 Gb was obtained from the aforementioned samples. The average error rate for each group of samples was less than 0.02%. Q30 values were observed to be 94.76%, 95.25%, 95.25%, and 95.28% for each respective group, while the GC content ranged from 50.18% to 52.31% (Supplementary Table S2). Alignment analysis showed that more than 95.00% of reads from each sample aligned with the reference genome, and additionally, 76.92%–87.17% of reads specifically aligned to unique positions within the reference genome (Supplementary Table S2), indicating high confidence in the transcriptome sequencing results in this study.

### Transcription functional annotation of gonadal transcriptome

**FIGURE 3:**
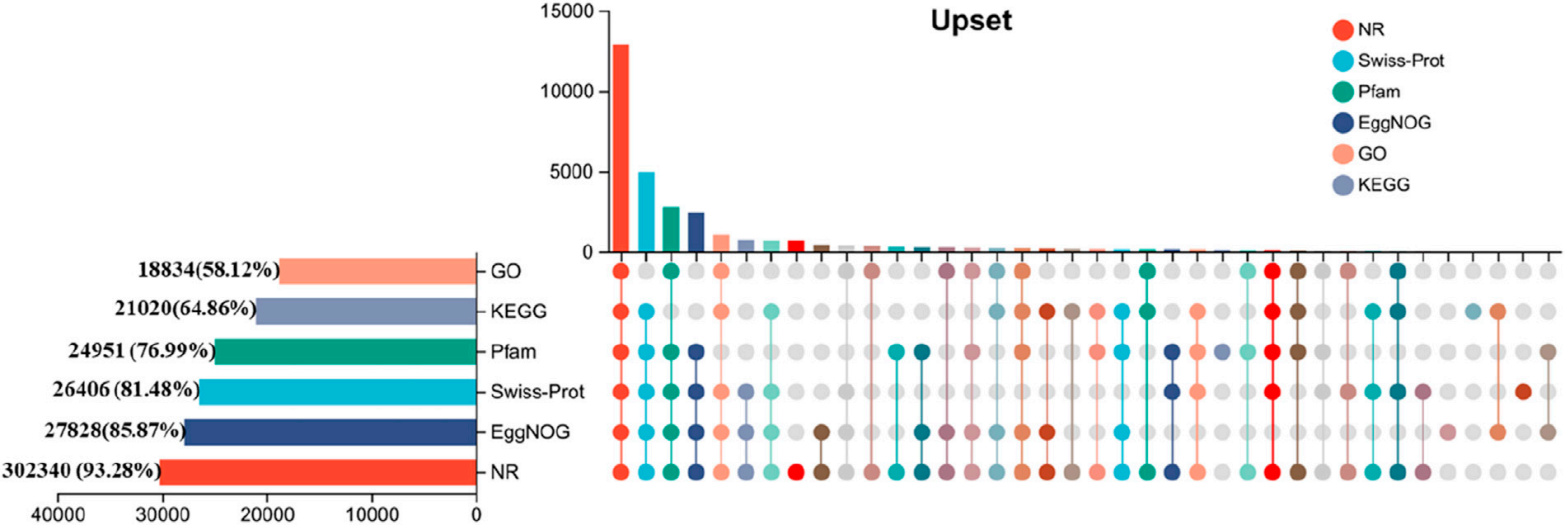
Functional annotation and statistics of DEGs in the gonads of LMB.

The analysis of transcription length distribution revealed that there were 17,257 unigenes (20.46%) in the range of 0-1000bp, 14,439 unigenes (17.12%) in the range of 1,000-1800bp, and 52,658 unigenes larger than 1800bp (62.43%) (Supplementary Figure S1). A total of 30,343unigenes (93.63%) matched the public database. Among them, six databases including GO, KEGG, COG, NR, Swiss-Prot and Pfam annotated to a total of 18,834 (58.12%), 21,020 (64.86%), 27,828 (85.87%), 302,340 (93.28%), 26,406 (81.48%) and 24,951 (76.99%) genes respectively (Figure 3).

### Screening and qRT-PCR validation of sex genes

**FIGURE 4:**
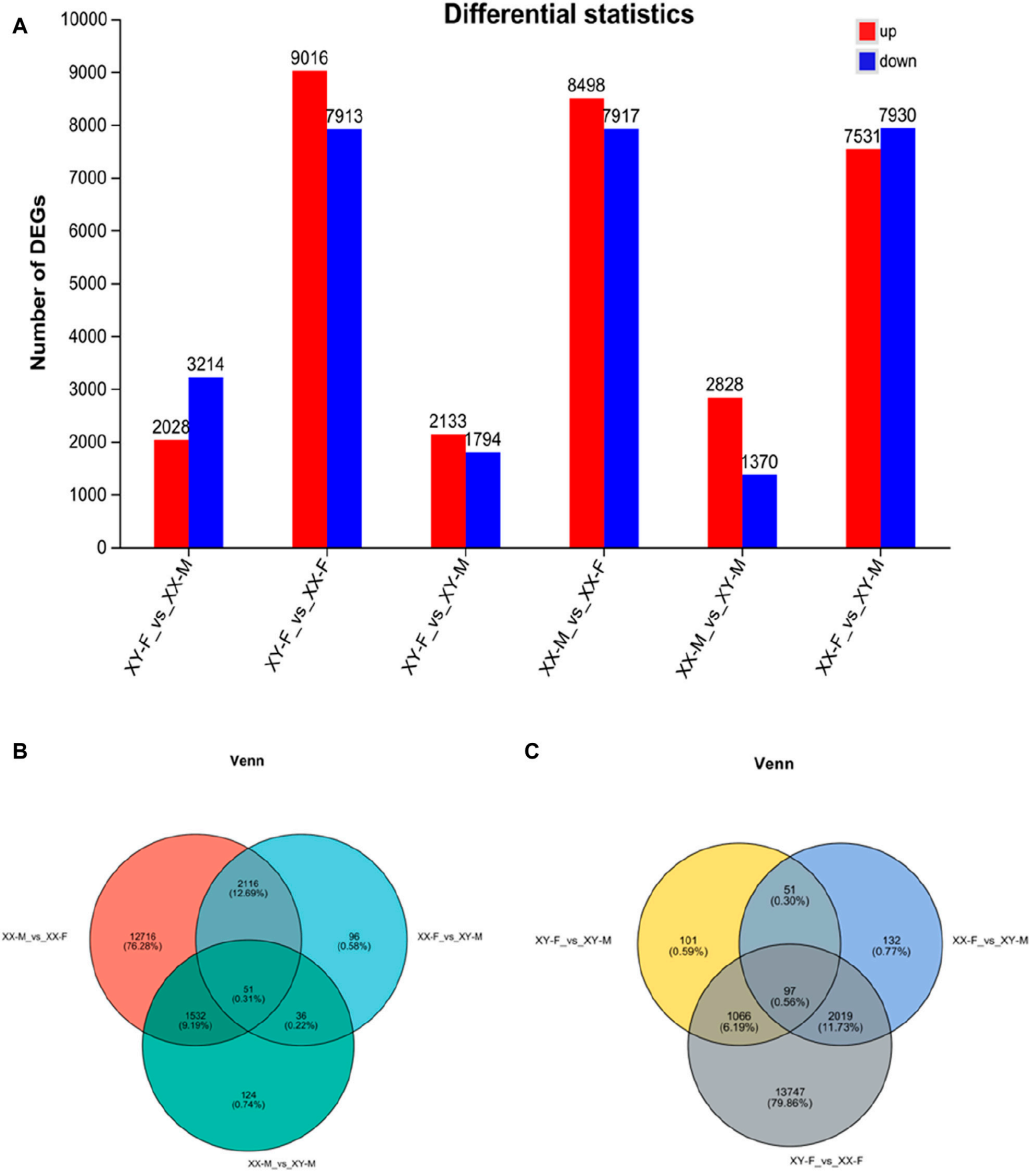
The number of DEGs number in bar chart and venn diagram in the different groups. **(A)**: The number of DEGs in the gonads of LMB in different groups. **(B)**: DEGs number and Venn diagram of overlap of the XX-M_vs._XX-F, XX-M_vs._XY-M and XX-F_vs._XY-M. **(C)**: DEGs number and Venn diagram of overlap of the XY-F_vs._XX-F, XY-F_vs._XY-M and XX-F_vs._XY-M.

DESeq2 software was used to screen DEGs with p-adjust<0.05 and |Log_2_(Fold Change) |>1 as the criteria. A total of 2,753 DEGs were found among XX-M, XY-F, XY-M and XX-F. In XX-M_vs._XX-F, XY-F_vs._XY-M, XX-M_vs._XY-M, XY-F_vs._XX-F, XX-F_vs._XY-M and XY-F_vs._XX-M, there were 8,498genes, 689 genes, 1,287 genes, 9,016 genes, 196 genes and 2,028 genes upregulated, respectively, meanwhile, there were 7,917 genes, 626 genes, 456 genes, 7,193 genes, 2,102 genes, 31,214 genes were downregulated, respectively (Figure 4A). Further analysis found 51 DEGs in XX-M_vs._XY-M, XX-M_vs._XX-F and XY-M_vs._XX-F (Figure 4B). And there are 97 DEGs in the XY-F_vs._XY-M, XY-F_vs._XX-F, and XY-XX-M_vs._XX-F (Figure 4C).

**FIGURE 5:**
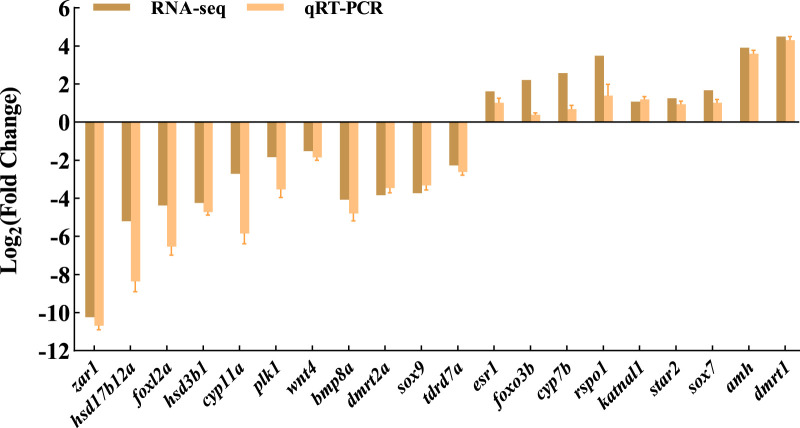
Verification of the expression in RNA-seq and qRT-PCR.

To validate the transcriptome results, a total of 20 DEGs with significantly different expression levels from the control group were selected for qRT-PCR analysis. These DEGs encompassed various biological pathways and processes, including steroid hormone biosynthesis (*hsd3b1*, *cyp7b*, *hsd17b12a*, and *cyp11a*), ovarian steroidogenesis pathway (*star2*), wnt signaling pathway (*rspo1* and *wnt4*), cAMP signaling pathway (*sox9* and *amh*), foxo signaling pathway (*foxo3b* and *plk1*), TGF-beta signaling pathway (*bmp8a*), estrogen signaling pathway (*esr1*), as well as other genes related to sex determination and differentiation (*dmrt2a*, *dmrt1*, *tdrd7a*, *zar1*, *foxl2a*, *katnal1*, and *sox7*). The expression levels of qRT-PCR results are consistent with RNA-seq, but the relative expression quantities are not the same (Figure 5).

### GO analysis of DEGs

**FIGURE 6:**
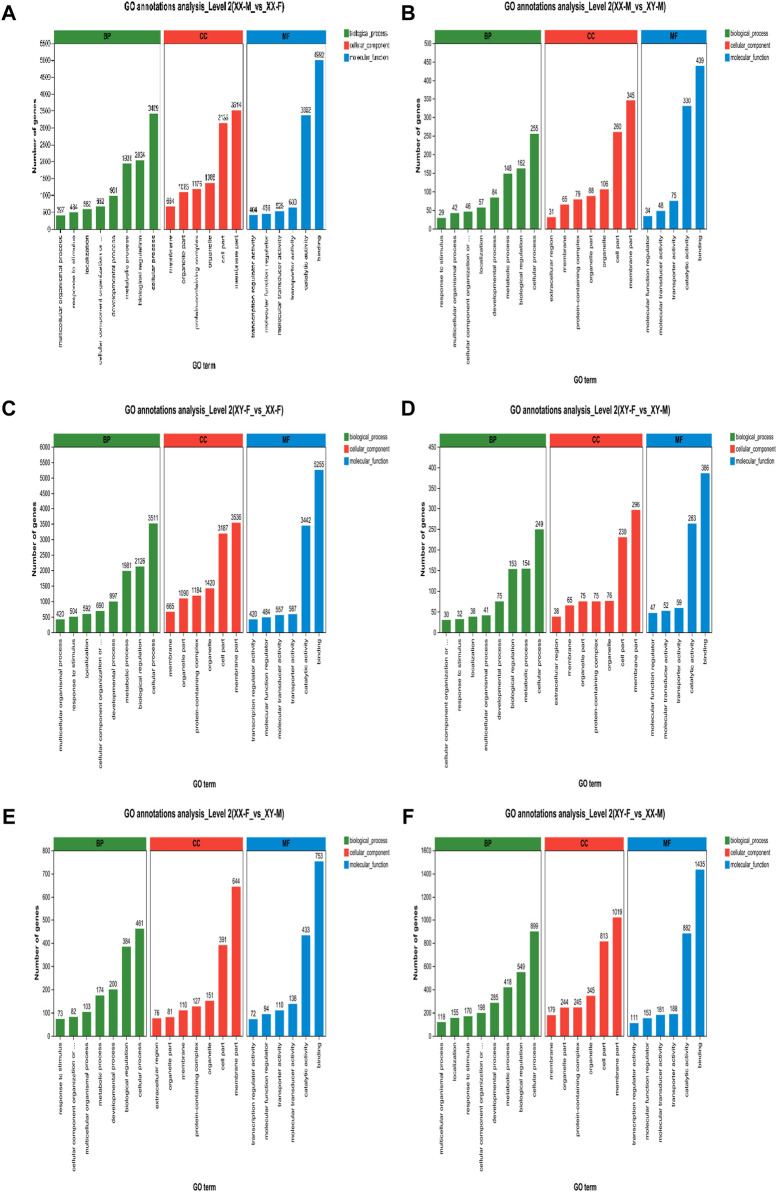
GO annotation enrichment statistics scatter plot of differentially expressed genes of LMB. **(A)**: XX-M_vs._XX-F; **(B)**: XX-M_vs._XY-M; **(C)**: XY-F_vs._XX-F; **(D)**: XY-F_vs._XY-M; **(E)**: XX-F_vs._XY-M; **(F)**: XY-F_vs._XX-M.

In this study, we analyzed the GO function of XX-M, XY-F, XY-M and XX-F. The results are presented in Table 2 and Figure 6. Bar charts depict the top 20 significantly enriched GO items (Figure 6). These include tRNA metabolic process (GO:0006399), dynein complex (GO:0030286), ncRNA metabolic process (GO:0034660), intermediate filament (GO:0005882), enteric nervous system development (GO:0048484), meiotic cell cycle process (GO:1903046), reproductive process (GO:0022414) and steroid biosynthetic process (GO:0006694) as shown in Supplementary Figure S2.

**TABLE 2:** The DEGs of GO analysis.

Groups	Biological process	Cellular component	Molecular function	Total DEGs
XX-M_vs._XX-F	10,485	10,938	10,399	31,292
XX-M_vs._XY-M	823	974	926	2,723
XY-F_vs._XX-F	10,821	11,082	10,158	32,061
XY-F_vs._XY-M	772	855	807	2,434
XX-F_vs._XY-M	1,477	1,578	1,600	4,657
XY-F_vs._XX-M	2,792	2,845	2,950	8,587

**FIGURE 7:**
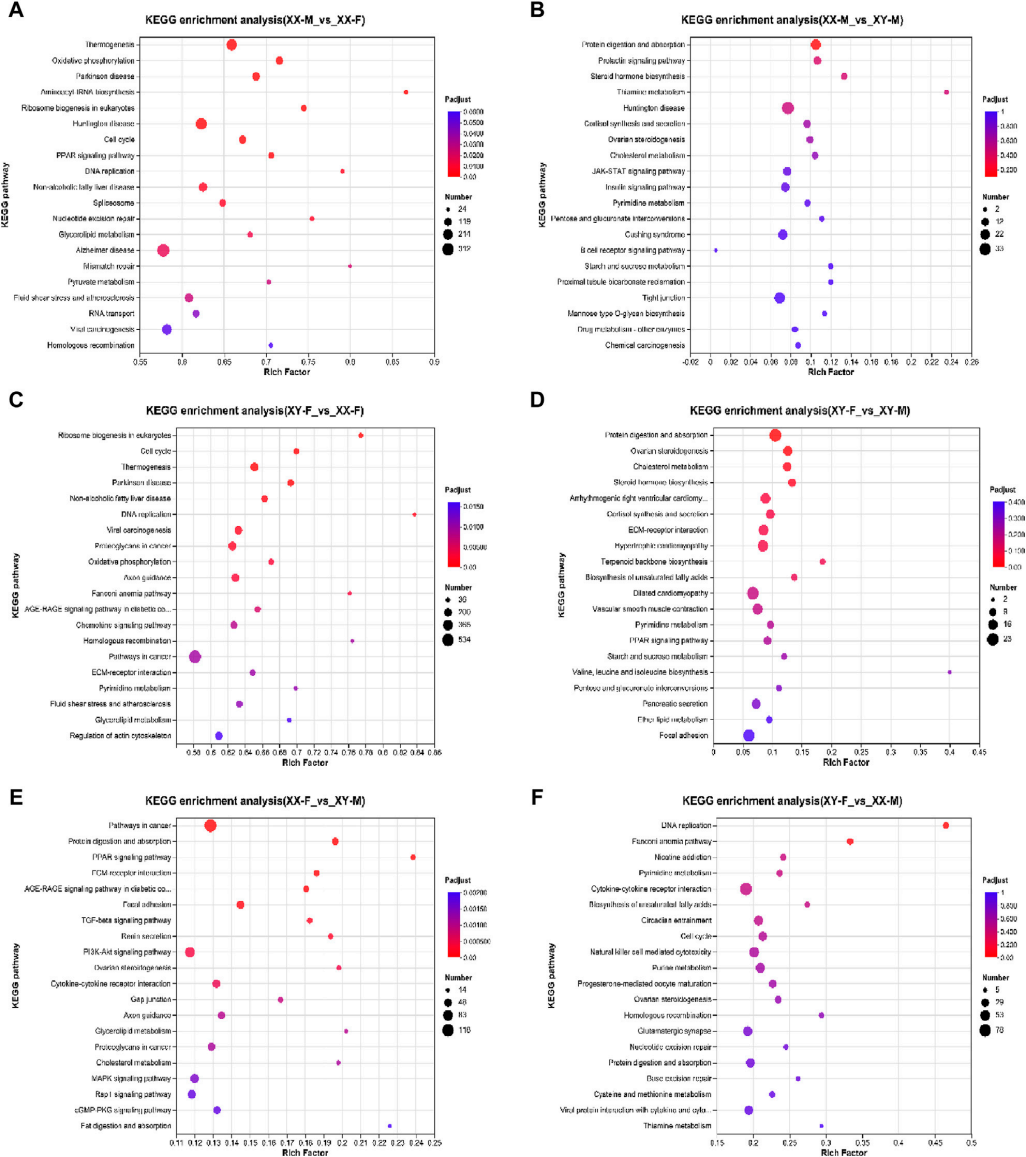
KEGG functional enrichment statistics scatter plot of differentially expressed genes of LMB. **(A)**: XX-M_vs_XX-F; **(B)**: XX-M_vs_XY-M; **(C)**: XY-F_vs_XX-F; **(D)**: XY-F_vs_XY-M; **(E)**: XX-F_vs_XY-M; **(F)**: XY-F_vs_XX-M

**FIGURE 8:**
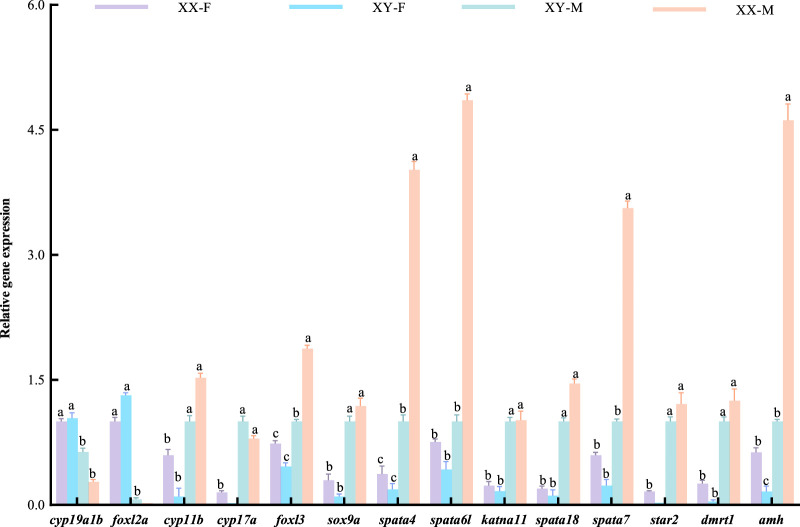
The expression patterns of 14 genes in XX-F, XY-F, XY-M, XX-M.

KEGG pathway analysis was conducted to explore the metabolic pathway enriched in different comparison groups of DEGs. The results showed that XX-M_vs._XX-F, XX-M_vs._XY-M, XY-F_vs._XX-F, XY-F_vs._XY-M, XX-F_vs._XY-M and XY-F_vs._XX-M were associated with 347, 313, 347, 306, 321 and 337 known KEGG pathways respectively. A scatter diagram was used to illustrate the top 20 KEGG pathways (*p* < 0.05) (Figure 7). Through KEGG enrichment analysis, several sex-related signaling pathways were identified including steroid hormone biosynthesis, wnt signaling pathway, oocyte meiosis, estrogen signaling pathway and gonadotropin secretion pathway. In total, there were 57 sex-related DEGs identified (Supplementary Table S3), among which 12 were associated with male sex reversal and 2 were related to female sex reversal (Table 3). The expression patterns of the 14 candidate genes confirmed the findings of the RNA-seq data in XX-F, XY-F, XY-M and XX-M groups (Figure 8).

**TABLE 3:** Genes associated with sex reversal in LMB.

Gene name	Description	Function	XX-M_vs._XX-F regulate	XY-F_vs._XY-M regulate	Sex-bias
*star2*	steroidogenic acute regulatory protein 2	steroids biosynthetic process	up	down	male
*cyp17a*	steroid 17-alpha-hydroxylase/17,20 lyase	androgen biosynthetic process	up	down	male
*cyp11b1*	cytochrome P450 11B, mitochondrial	Steroid hormone biosynthesis	up	down	male
*dmrt1*	double sex and mab-3 related transcription factor 1	male sex determination	up	down	male
*amh*	anti-Mullerian hormone	sperm capacitation, spermatogenesis	up	down	male
*sox9a*	SRY-box transcription factor 9a	male gonad development	up	down	male
*katnal1*	katanin p60 subunit A-like 1	spermatogenesis	up	down	male
*spata4*	spermatogenesis associated 4	spermatogenesis	up	down	male
*spata6l*	spermatogenesis associated 6	spermatogenesis	up	down	male
*spata7*	spermatogenesis associated 7	spermatogenesis	up	down	male
*spata18*	spermatogenesis associated 18	spermatogenesis	up	down	male
*foxl2a*	fork head box L2-like	estrogen response element binding	down	up	female
*foxl3*	fork head box L3	gonadal development	up	down	male
*cyp19a1b*	brain aromatase	steroid biosynthetic process	down	up	female

## Discussion

### Effects of exogenous hormones on early gonad development of LMB

Previous studies have generally suggested that fish exhibit a relatively limited degree of evolutionary plasticity, making their early sex determination and differentiation susceptible to environmental influences. It has been documented that successful induction of sex transformation has been achieved in 47 species of fish (Mei and Gui, 2015). For instance, in cases of undifferentiated gonads, exogenous male or female hormones can induce the development of either testes or ovaries from primary gonads in *O. niloticus* (Chen et al., 2016). In bluegill sunfish (*Lepomis macrochirus*) and rainbow trout (*O. mykiss*), 17β-E_2_ or17α-MT produced pseudo-female and pseudo-male populations, respectively (Wang, et al., 2008; Xu, et al., 2021). In this study, the treated groups obtained XX-M and XY-F fish, consistent with previous results (Zhou, et al., 2021; Du et al., 2022). The gonad structure of XX-M was similar to XY-M, including spermatogonia and spermatocytes; and the structure of XY-F was similar to XX-F, such as primary oocytes. Moreover, the levels of E_2_ and T in serum increased after the treatments, it was consistent with the conclusion that exogenous hormones directly affect gonadal differentiation and upregulate E_2_ or T hormone levels *in vivo* (Cavaco et al., 2001; Labadie and Budzinski, 2006). These results indicated that exogenous hormones played an important role in the process of sex reversal in fish.

### Changes of sex-related signaling pathways in LMB during sex reversal

By comparing the results of gonadal transcriptome, the steroid hormone biosynthesis pathway is significantly enriched in all comparison groups. Consider discussing the changes in this pathway-related genes to ensure a more complete understanding of the possible mechanisms of gender reversal in this study. Sex steroid hormones play a crucial role in promoting gonad development and maintaining secondary sex characteristics (Kinsley et al., 2012). The synthesis of fish sex steroid hormones involves cholesterol as the precursor, and the synthesis of androgens or estrogens is completed under the synergistic regulation of transport enzymes, lyases and hydrogenases (such as *star*, *cyp11a/17a* and *17β-hsd*) (Abaffy et al., 2023). In mammals, cholesterol transport into mitochondria by *star* is crucial for synthesizing sex steroid-like hormones (Rone et al., 2009). In fish, *star* is divided into two subtypes, namely, *star1* and *star2*. While *star1* is mainly expressed in the head kidney tissue and involved in cortisol production, *star2* is primarily expressed in the gonadal tissue and is involved in the production of male hormones (Yu et al., 2014). In the sex steroid hormone synthesis pathway, estrogen binding to estrogen receptors leads to a downregulation of the *star2* expression, thereby reducing the synthesis of male hormones (Strauss et al., 2009). In this study, 17α-MT treatment significantly upregulates *star2* expression, while 17β-E_2_ treatment markedly downregulates it, suggesting that *star2* is biased toward male gonad differentiation and development in LMB. Furthermore, its downstream gene *cyp11a* potentially influences spermatophore development and shows specific expression in olive flounder (*Paralichthys olivaceus*) (Liang et al., 2018) and brown hagfish (*Paramyxine atami*) (Nishiyama et al., 2015). In this study, both 17α-MT and 17β-E_2_ treatments significantly downregulated the expression of *cyp11a*, indicating its involvement in the sexual differentiation process of LMB and its bias towards the differentiation and development of testes. Moreover, *cyp11a* is highly susceptible to negative feedback regulation. On the other hand, *cyp17a* within its family is involved in ovarian differentiation and development. Knocking out *cyp17a* in *Danio rerio* resulted in all mutants developing into males (Xiong et al., 2015). Yellowfin seabream (*A. latus*), a hermaphroditic fish species, undergoes gradual transformation of testes into ovaries at 2-year-old. Analysis of the gonadal transcriptome in 2-year-old *Acanthopagrus latus* revealed a significantly upregulated of *cyp17a* expression is during the transition from sperms to ovaries (Li et al., 2020). In contrast, the results of this study showed that *cyp17a* expression was downregulated in 75 dph LMB, likely due to its correlation with gonad development. Meanwhile, *17β-hsdb1* is mainly expressed in mammalian ovaries and plays a crucial role in converting estrone to estradiol (Lukacik et al., 2006; Lai et al., 2022). Similarly, teleost fish like *O*. *niloticus*, also exhibits significant sexual dimorphism for *17β-hsdb1* (Zhou et al., 2005). In this study, 17α-MT treatment significantly downregulated the expression of *17β-hsdb1*, suggesting that *17β-hsdb1* may be involved in early gonadal development of LMB females. In conclusion, the steroid synthesis pathway plays an important role in the gender reversal process of LMB and warrants further investigation.

### Changes of sex-related genes in LMB during sex reversal

In order to understand the changes of genes related to sex reversal in LMB, we analyzed the expression of several candidate genes in XY-F and XX-M, which were consistent with XX-F and XX-M, respectively, including key male sex reversal genes, such as *dmrt1*, *amh*, *sox9a*, *katnal1*, *spata4/6L/7/18*, *foxl3*; as well as female sex reversal genes, like *foxl2a* and *cyp19a1b*. The processes of sex determination and differentiation are complex biological phenomena that involve the regulation of multiple genes. In mammals, *dmrt1* plays a pivotal role in the development and maintenance of male testis (Zarkower and Murphy, 2022). Following *dmrt1* knockout, mice (*Mus musculus*) experience degeneration and loss of reproductive function in their testes (Inui et al., 2017). In teleost fish, it has been confirmed that *dmrt1* is crucial for fish spermatogenesis. Male *D*. *rerio* with deleted *dmrt1* exhibit abnormalities in spermatogenesis and infertility, which can lead to male sex reversal (Herpin and Schartl, 2011; Webster et al., 2017). Intraperitoneal injection of *dmrt1* recombinant protein significantly increases the levels of testosterone and 11-ketol-testosterone on *P*. *olivaceus*, resulting in a male ratio increased to 82%. This indicated that *dmrt1* is involved in flounder sex reversal (Shu et al., 2022). Transcriptome sequencing of gonad and somatic tissues by Guan et al. (2022) revealed an important role of *dmrt1* gene in sex determination of LMB. In this study, 17α- MT treatment significantly upregulated the expression of *dmrt1* in female LMB, while 17β-E_2_ treatment downregulated the expression of *dmrt1* in male LMB. Moreover, the results of tissue sections and testosterone levels were consistent with those of previous studies, confirming that *dmrt1* played an important role in the process of gender conversion from female to male in LMB. The downstream genes of *dmrt1*, *amh* and *sox9a*, are involved in spermatogenesis and male gonad development (Sudbeck et al., 1996; Sinisi et al., 2003). In this study, *amh* and *sox9a* were significantly upregulated after 17α-MT treatment, indicating that high expression of *dmrt1* can activate downstream genes related to male development pathways in undifferentiated LMB and promote gonadal-to-testis differentiation. In addition, Zheng et al. (2021) found through transcriptome sequencing that related genes promoting spermatogenesis during sexual reversal of *Pseudobagrus ussuriensis*, such as the *katnal1* and *spata* families, were significantly upregulated. In this study, the expressions of other *katnal1* and *spata4/6l/7/18* were also significantly upregulated after 17α-MT treatment, while downregulated after 17β-E_2_ treatment. This suggests that the *katnal1* and *spata* families participated in and assisted female fish in with gender reversal in pseudo-male fish. *Foxl2a*, a subtype of *foxl2*, regulates aromatase expression and is involved in ovarian differentiation and follicle development (Caulier et al., 2015). In this study, *foxl2a* was upregulated in the 17α-MT treatment group and downregulated after 17β-E_2_ treatment, suggesting that *foxl2a* plays an important role in early ovarian development. Unlike *foxl2*, its homologous copy *foxl3* is not only involved in ovarian differentiation but also related to testis development. In *O*. *latipes*, *foxl3* is exclusively expressed in female germ cells, while the absence of *foxl3* leads to promote sperm cells development occurring in the gonads of female individuals (Kikuchi et al., 2020). In *O*. *niloticus*, *foxl3* is associated with female sex determination (Dai et al., 2021). However, 17α-MT significantly upregulated *foxl3* expression during sex reversal in *Siniperca chuatsi* (Zhu et al., 2022) and *E*. *coioides* (Lyu et al., 2019). In the study, *foxl3* was found to be upregulated in the 17α-MT treated group and downregulated after 17β-E_2_ treated, suggesting that *foxl3* is biased towards testis development in LMB. During the early development of bony fish, *cyp19a1b* can catalyze the production of gonadal estrogen and is highly sensitive to estrogen. Its expression level is closely related to estrogen homeostasis, which significantly regulates the sex differentiation and neuroendocrine functions of fish (Sawyer et al., 2006; Le Page et al., 2011; Diotel et al., 2013; Alharthy et al., 2017).

## Conclusion

In this study, we conducted a comprehensive analysis of the gonadal transcriptomes in LMB subjected to 17α-MT or 17β-E_2_ treatment for the first time. The findings revealed striking similarities in gene expression patterns between pseudo-female or pseudo-male fish and their normal female or male counterparts. A total of 12 DEGs associated with male sex reversal were identified: *star2*, *cyp17a*, *cyp11b1*, *dmrt1*, *amh*, *sox9a*, *katnal1*, *spata4*, *spata6l*, *spata7*, *spata18* and *foxl3*. Additionally, 2 DEGs linked to female sex reversal were identified: *foxl2a* and *cyp19a1b*. These gene expression changes affect the direction of sex differentiation in LMB. *dmrt1* and *cyp19a1b* with significant expression changes may play an important role in the process of sex reversal in LMB.

## Data Availability

The data presented in the study are deposited in the GenBank of NCBI (https://www.ncbi.nlm.nih.gov/), the SRA login number is PRJNA1060465.
