# Supplementary figures and images for: Transcriptome analysis of four types of gonadal tissues in largemouth bass (Micropterus salmoides) to reveal its sex-related genes
Source: Front Genet. 2024 Aug 26;15:1459427. doi: 10.3389/fgene.2024.1459427 (PMC11381392; doi:10.3389/fgene.2024.1459427)

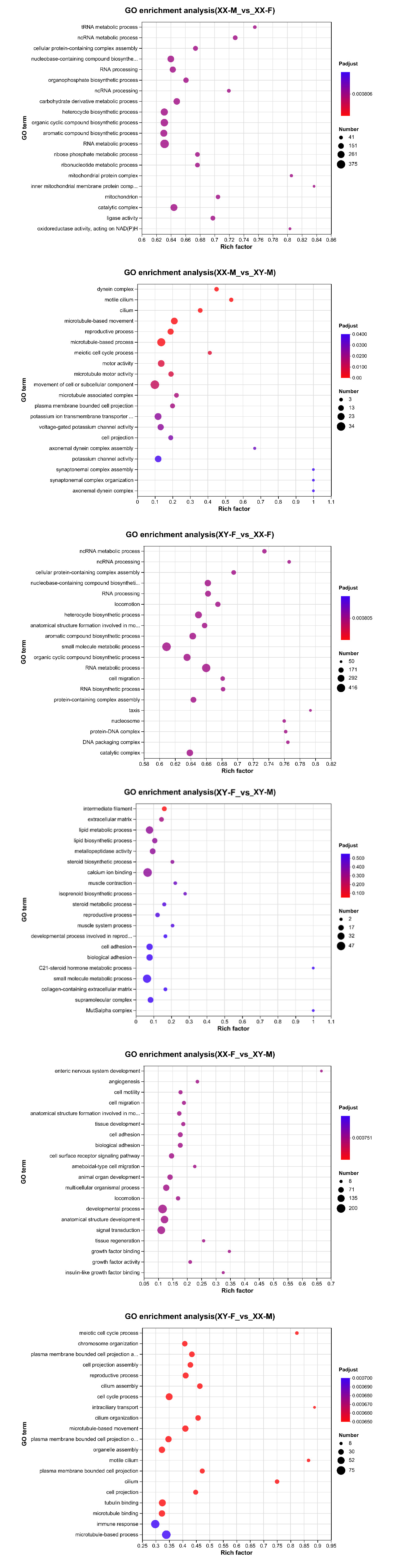

Supplement: Supplementary file 1 [file Image2.jpg]

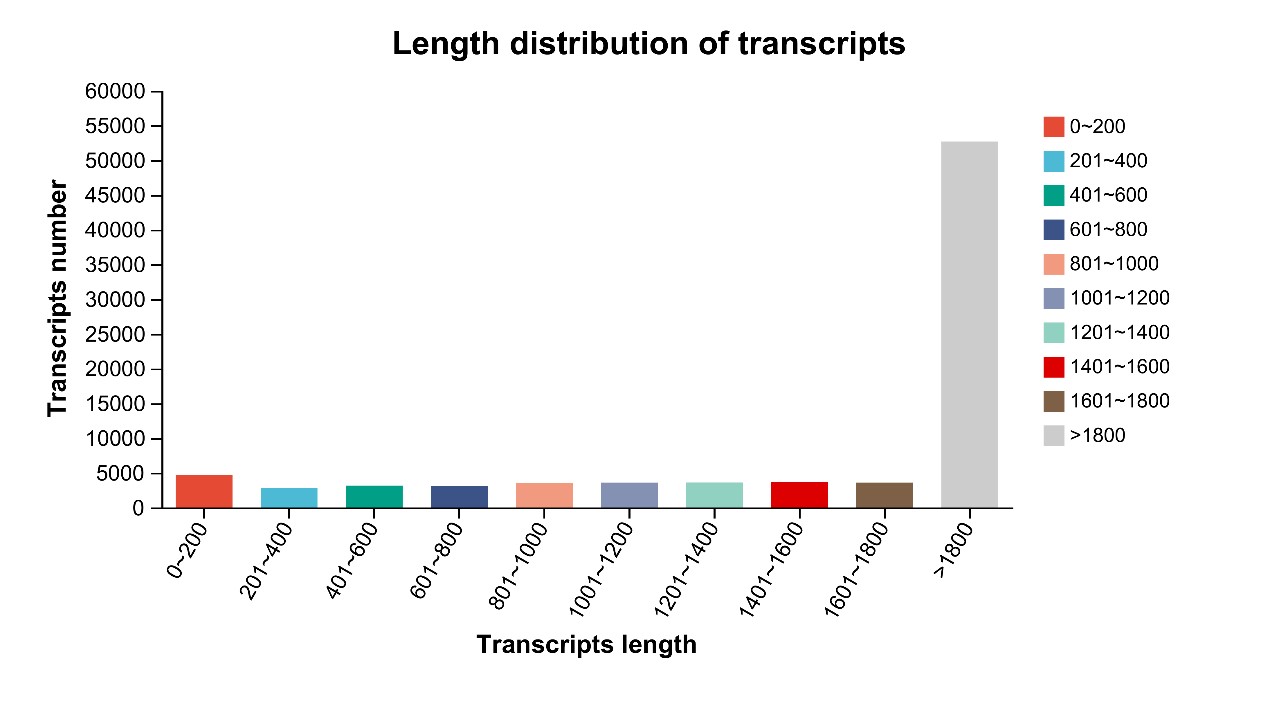

Supplement: Supplementary file 4 [file Image1.jpg]
