# Supplementary material for: Transcriptome analysis of four types of gonadal tissues in largemouth bass (Micropterus salmoides) to reveal its sex-related genes
Source: Front Genet. 2024 Aug 26;15:1459427. doi: 10.3389/fgene.2024.1459427 (PMC11381392; doi:10.3389/fgene.2024.1459427)
Supplement: Supplementary file 2 [file Table1.docx]

Table S1. Primers of different expression genes used for the qRT-PCR analysis

| gene | forward primer sequence(5’- 3’) | reverse primer sequence(5’- 3’) |
| --- | --- | --- |
| *star2* | TCTGCTGTGGAATCTCCTACCC | GAGCCTTTCTCATTGCTTCTTG |
| *cyp17a* | GTGGCAAAGGACAGCCTGGT | GCTCTCAGCAGAGCGTCCAG |
| *cyp11b* | TCCTTGGAGTCGCAGAAGTT | GAAGATGTGGTCCCATGCAC |
| *dmrt1* | GAACCACGGCTATGTGTCTCCT | AATCCCAAGTTCCTCCTCCTGA |
| *amh* | CCAGTTGGAGAAGGAGAAGAAGGT | CTGGGGAAACAGGAACTGATACAA |
| *sox9a* | GGTCCGGTTCGGACACTGAG | GCGTCCAGTCGTAGCCCTTC |
| *katnal1* | AGCCAGTTGACATCCTTGCC | AACCCCTTTCTTTCCCTTATCA |
| *spata4* | AGCTGTTGCGGCTCGTTACT | AGTCGAGGCTTTGCAGCCAT |
| *spata6l* | GCAGACCCAGGACCTCCTCT | GGTGTTGCAGAACCCAGGCT |
| *spata7* | GCCCTTTCTGCCCTCCATCC | GTGGGCTGACTGAGGACGAC |
| *spata18* | TGCCTAGGCTCAGTCGCTCT | AGCGTCGAAGCAGGTTCTGG |
| *foxl3* | ACAGGGTGTTGCTGCTGGAC | GCTGTACGCGGGTCTACACA |
| *foxl2a* | CGGAGGATGACGCAATGG | GGTGGTTTCTGGGACGGA |
| *cyp19a1b* | GCCATCCTAGTCGCTCTGTTGTC | TGTTGGTCTGCCTGATGCTGTTG |
| *β-actin* | AAAGGGAAATCGTGCGTGAC | AAGGAAGGCTGGAAGAGGG |
