# Supplementary material for: Transcriptome analysis of four types of gonadal tissues in largemouth bass (Micropterus salmoides) to reveal its sex-related genes
Source: Front Genet. 2024 Aug 26;15:1459427. doi: 10.3389/fgene.2024.1459427 (PMC11381392; doi:10.3389/fgene.2024.1459427)
Supplement: Supplementary file 3 [file Table2.docx]

Table S2 Transcriptome library sequencing and statistics data of gonads in LMB*.*

| Sample | Total reads | Raw  reads | Raw  bases | Clean  reads | Clean  bases | Error  rate(%) | Q30  (%) | GC  content(%) | Total  mapped | Multiple  mapped | Uniquely  mapped |
| --- | --- | --- | --- | --- | --- | --- | --- | --- | --- | --- | --- |
| XX-M3 | 46,284,712 | 47,729,468 | 7,207,149,668 | 46,284,712 | 6,619,697,210 | 0.02 | 95.34 | 50.62 | 44,567,702(96.29%) | 4,285,114(9.26%) | 40,282,588(87.03%) |
| XX-M2 | 45,740,090 | 46,425,932 | 7,010,315,732 | 45,740,090 | 6,745,174,341 | 0.03 | 93.66 | 49.7 | 43,998,395(96.19%) | 4,488,500(9.81%) | 39,509,895(86.38%) |
| XX-M1 | 55,011,376 | 56,499,530 | 8,531,429,030 | 55,011,376 | 7,972,203,501 | 0.02 | 95.29 | 50.23 | 52,921,108(96.2%) | 4,457,203(8.1%) | 48,463,905(88.1%) |
| XY-F3 | 52,512,510 | 54,318,668 | 8,202,118,868 | 52,512,510 | 7,587,480,996 | 0.02 | 95.1 | 50.34 | 50,519,614(96.2%) | 5,171,377(9.85%) | 45,348,237(86.36%) |
| XY-F2 | 52,153,400 | 53,519,860 | 8,081,498,860 | 52,153,400 | 7,562,464,444 | 0.02 | 95.45 | 50.37 | 50,148,058(96.15%) | 5,331,304(10.22%) | 44,816,754(85.93%) |
| XY-F1 | 58,896,884 | 60,786,756 | 9,178,800,156 | 58,896,884 | 8,545,554,090 | 0.02 | 95.2 | 50.25 | 56,766,115(96.38%) | 5,131,295(8.71%) | 51,634,820(87.67%) |
| XY-M3 | 53,217,868 | 54,027,580 | 8,158,164,580 | 53,217,868 | 7,697,875,177 | 0.02 | 95.13 | 52.27 | 50,442,117(94.78%) | 10,061,322(18.91%) | 40,380,795(75.88%) |
| XY-M2 | 41,930,744 | 43,411,376 | 6,555,117,776 | 41,930,744 | 6,022,230,220 | 0.02 | 95.31 | 50.34 | 40,053,289(95.52%) | 3,505,153(8.36%) | 36,548,136(87.16%) |
| XY-M1 | 54,920,386 | 56,217,656 | 8,488,866,056 | 54,920,386 | 7,915,595,510 | 0.02 | 95.32 | 50.16 | 52,615,222(95.8%) | 4,405,016(8.02%) | 48,210,206(87.78%) |
| XX-F3 | 55,913,414 | 56,721,320 | 8,564,919,320 | 55,913,414 | 8,072,465,161 | 0.02 | 95.13 | 52.44 | 52,903,059(94.62%) | 9,682,407(17.32%) | 43,220,652(77.3%) |
| XX-F2 | 55,343,088 | 56,038,256 | 8,461,776,656 | 55,343,088 | 7,995,349,582 | 0.02 | 95.48 | 52.18 | 52,772,818(95.36%) | 10,048,384(18.16%) | 42,724,434(77.2%) |
| XX-F1 | 48,418,678 | 49,018,964 | 7,401,863,564 | 48,418,678 | 7,021,426,893 | 0.02 | 95.23 | 52.31 | 46,028,620(95.06%) | 9,110,683(18.82%) | 36,917,937(76.25%) |
